# 

**Published:** 1858-10-01

**Authors:** 


### INDEX TO VOL. XI.

Arlidge, Dr., on the asylums of France, 59
Asylums of France, by Dr. Arlidge, 59
——— in Norway, by Dr. L. Lindsay, 246
——— private, popular outcry against, xxxiii.
Autobiography of a drunkard, 461
Auzony, M., on disordered sentiments and affections, 661

Body *v.* Mind, 1
——— and Mind, by Dr. Jamieson, 335
Brontë, Charlotte, 295

Civilization, on, 176
Cysticerci in the brain: singular case of insanity, 645
Czermack, Dr., on a singular case of insanity, 645

Deaf and Dumb, responsibility and legal relations of, by Dr. Jendritza, 643
Dipsomania, on, by Dr. D. Skae, 349
Drunken insanity, on, 349
Drunkard, autobiography of a, 461
Dunn, Mr., on physiological psychology, 317
Dreams, on a particular class of, induced by food, 567

Earle, Dr., on the paralysis of the insane, 444

Ferrus and Garnier, MM., on somnambulism and extraordinary neuroses, 652
Follet, Dr., on lesions of the relative functions, 647
Foreign Psychological Literature, 640

Garnier and Ferrus, MM., on somnambulism and extraordinary neuroses, 652
Gray, Dr., on homicide in insanity, 142

Homicide in insanity, by Dr. Gray, 142
Hood, Dr., on the pathology of insanity, 97
Hoppus, Dr., on the psychology of Kant, 494
Howe, Dr., on the causes of idiocy, 365

Idiocy, causes of, by Dr. Howe, 365
Indian rebellion, moral and psychological aspects of, 39
Insane, condition of, 88
——— criminal responsibility of, 166
——— paralysis of, by Dr. Earle, 444
Insanity, pathology of, by Dr. Hood, 97
——— intemperance as a form of, 102
——— homicide in, by Dr. Gray, 142
——— treatment of, in Julius Hospital, Würzburg, 124
——— Dr. von Marcus on, 124
——— Dr. Ernst Schmidt on, 124
——— legal doctrine of responsibility in cases of, connected with alleged criminal acts, by Dr. Forbes Winslow, 214
——— in Norway, by Dr. L. Lindsay, 246
——— drunken, or dipsomania, by Dr. D. Skae, 349
——— tendency of misdirected education to produce, by Dr. Jarvis, 424
——— a singular case of, 490
——— physical symptoms of, by M. Sauze, 641
——— singular case of, by Dr. Czermack, 645
Intemperance as a form of mental disorder, 102

Jamieson, Dr., on mind and body, 335
Jarvis, Dr., on the tendency of misdirected education and the unbalanced mind to produce insanity, 424
Jendritza, Dr., on the responsibility and legal relations of the deaf and dumb, 643
Juridical Society and the criminal responsibility of the insane, 166

KANT, psychology of, by Professor Hoppus, 494

LANGUAGE of orators, poets, &c., psychological basis of, 567
Leach, Rev. W. J. J., case of, 668
Lindsay, Dr. W. Lauder, on insanity and lunatic asylums in Norway, 246
London, on the moral pathology of, 539
Lunacy legislation, 523
——— present state of, in England and Wales, 626
——— Commissioners in, report of, 626
——— Commission in, Case of Mr. Leach, 668
——————— Mr. Ruck, xxx.
——————— Mrs. Turner, xxx.

MANIA with homicidal tendency, case of, by M. Morel, 660
Marcus, Dr. von, on insanity, 124
Mayo, Mr., on the psychological basis of the language of orators, poets, &c., 567
Mental disorder, intemperance considered as a form of, 102
Mind *v.* Body, 1
Mind and Body, by Dr. Jamieson, 335
Mind, unbalanced, tendency of, to produce insanity, by Dr. Jarvis, 424
Morel, M., on a case of mania with homicidal tendency, 663
Mormonism, xxiii.

NERVOUS System, Dr. E. Brown-Séquard on, i.
Norway, insanity and asylums in, by Dr. L. Lindsay, 246

OXFORD examination of persons not members of the University, xxv.

PHANTASMATA, 136
Physiological psychology, by R. Dunn, F.R.C.S., 317
Psychological literature, foreign, 640
——————— Quarterly retrospect, xvii. *et seq.*

RELATIVE functions, on the obliteration and aberration of, by Dr. Follet, 647
Renaudin, E. M., on the pathogenic influence of loss of sleep, 665
REVIEWS :—
Dr. Sieveking on Epilepsy, 626
Drs. Bucknill and Tuke's Manual of Psychological Medicine, 635
Ruck, Mr., case of, xxx.

SAUZE, M., on the physical symptoms of insanity, 641
Schmidt, Dr. Ernst, on insanity, 124
Sentiments and affections, on disordered, by M. Auzony, 661
Séquard, Dr. E. Brown-, on the nervous system, i.
Skae, Dr. David, on dipsomania, 349
Sleep, on the pathogenic influence of loss of, by M. Renaudin, 665
Somnambulism and extraordinary neuroses, on, 652
Suicide, on, 396
Suicides, xxxv.

THELLUSSON, Peter, xxvii.
Tuke, the late Samuel, Esq., of York, 173
Turner, Mrs., case of, xxx.

VIRGIN, apparition of, at Lourdes, xxi.

WINSLOW, Dr. Forbes, on the legal doctrine of responsibility in cases of insanity, 214
——————— on lunacy legislation, 543
Witchcraft, modern belief in, xviii.
Wills, remarkable, xxvii.—xxix.

YOUNG, Brigham, xxiii.

END OF VOL. XI.

